# Notification List of Bacterial Strains Made Available by the United Kingdom National Collection of Type Cultures in 2021

**DOI:** 10.1128/mra.00357-22

**Published:** 2022-07-26

**Authors:** Jake David Turnbull, Jo Dicks, Sunita Gurung, Hannah McGregor, The NCTC 2021 Depositors Cohort, Sarah Alexander

**Affiliations:** a The National Collection of Type Cultures, Culture Collections, UK Health Security Agency, Colindale, London, United Kingdom; Indiana University, Bloomington

## Abstract

Here, we report on the 47 bacterial strains made available by the National Collection of Type Cultures in 2021, alongside a commentary on these strains and their significance.

## ANNOUNCEMENT

The National Collection of Type Cultures (NCTC) is composed of approximately 6,000 bacterial reference and type strains, of which many are of medical and veterinary importance. The NCTC is currently in its 103^rd^ year of operation and is hosted by the recently formed UK Health Security Agency (UKHSA).

Each year, bacterial strains are added to the collection for many reasons. These reasons include but are not limited to individuals who wish to share strains of diagnostic or industrial utility, veterinary and public health establishments who wish to share strains that carry emergent or important virulence factors or antibiotic resistance determinants, and researchers who deposit proposed type strains of novel taxa into the collection in order to validly describe them. Here, we describe the strains made available from the NCTC to the scientific community in 2021.

Forty-seven bacterial strains were made available to the scientific community by the NCTC in 2021, which are all listed in Table 1. Of these 47 strains, 17 strains constitute novel prokaryotic taxa, with the remaining 30 strains comprised of historic strains (*n* = 4), antimicrobial resistant (AMR) reference strains (*n* = 6), contemporary clinical and veterinary reference strains (*n* = 17), type strains not previously held by the NCTC (*n* = 2), and a laboratory reference strain (*n* = 1). The full details for each strain (e.g., citations for the full taxon descriptions, strain provenance, and validated growth conditions) can be obtained from the NCTC online catalogue.

**TABLE 1:** The 47 strains made available by the National Collection of Type Cultures in 2021^*a*^

NCTC accession identifier	Species name	Primary reference	Available whole-genome sequence data	Taxon rank
NCTC 14351	Acholeplasma equirhinis	14	GCF_017052655.1	sp. nov.
NCTC 14318	Eikenella exigua	6	GCF_008805035.1	
NCTC 14180	Eikenella halliae	15	GCF_001648475.1	
NCTC 14179	Eikenella longinqua	15	GCF_001648355.1	
NCTC 14359	Escherichia ruysiae	16	GCF_902498915.1	
NCTC 14309	Mycoplasma procyoni	14	GCF_017052595.1	
NCTC 14411	Oceanivirga miroungae	17	GCF_902505095.1	
NCTC 14075	Paraburkholderia agricolaris	18	GCF_009455635.1	
NCTC 14396	Pseudoxanthomonas winnipegensis	19	GCF_004283755.1	
NCTC 14454	Staphylococcus durrellii	20	GCF_015594545.1	
NCTC 14453	Staphylococcus lloydii	20	GCF_015775975.1	
NCTC 14363	Streptococcus caledonicus	21	PRJEB33418	
NCTC 14455	Streptococcus pacificus	22	GCF_016481305.1	
NCTC 14341	Streptococcus vicugnae	22	GCF_016461705.1	
NCTC 14410	Streptococcus zalophi	22	GCF_016481285.1	
NCTC 14393	Enemella dayhoffiae	1	GCF_002250625.1	gen. nov., sp. nov.
NCTC 14394	Enemella evansiae	1	GCF_002250705.1	
NCTC 14120	Shigella flexneri	3	ERS179715	Historic strain
NCTC 14123	Shigella sonnei	3	ERS222760	
NCTC 14146	Shigella flexneri	3	ERS179747	
NCTC 14154	Shigella sonnei	3	ERS179739	
NCTC 14376	Escherichia coli	4		AMR reference strain
NCTC 14377	Escherichia col	4		
NCTC 14378	Escherichia coli	4		
NCTC 14379	Escherichia coli	4		
NCTC 14381	Escherichia coli	4		
NCTC 14385	Clostridioides difficile	5	GCF_900696735.1	
NCTC 14169	Clostridioides difficile			Contemporary clinical isolate
NCTC 14174	Clostridioides difficile			
NCTC 14178	Auritidibacter ignavus	7	GCF_003185985.1	
NCTC 14181	Eikenella exigua	15	GCF_001648495.1	
NCTC 14457	Staphylococcus aureus	8		
NCTC 14458	Staphylococcus aureus	8		
NCTC 14459	Staphylococcus aureus	8		
NCTC 14460	Staphylococcus aureus	8		
NCTC 14461	Staphylococcus aureus	8		
NCTC 14462	Staphylococcus aureus	8		
NCTC 14464	Staphylococcus aureus	8		
NCTC 14465	Staphylococcus aureus	8		
NCTC 14481	Legionella wadsworthii			
NCTC 14405	Neisseria animaloris	10		Contemporary veterinary isolate
NCTC 14406	Neisseria animaloris	10		
NCTC 14407	Neisseria animaloris	10		
NCTC 14408	Neisseria animaloris	10		
NCTC 14201	Ideonella sakaiensis	11	GCF_001293525.1	Type strain
NCTC 14293	Varibaculum cambriense	12		
NCTC 14582	Escherichia coli			Laboratory reference strain

a The 47 strains include type strains of 17 novel taxa and 30 additional reference and type strains from established taxa. The whole-genome sequence data are listed as publicly available whole-genome assemblies in NCBI RefSeq, with the exception of NCTC 14363 for which several contig-level assemblies exist at time of writing, and NCTC 14120, NCTC 14123, NCTC 14146, and NCTC 14154 for which Illumina short-read data exist at the time of writing. All whole-genome sequence data were provided by the depositors of each strain and are accessible via the NCBI using the above identifiers (https://www.ncbi.nlm.nih.gov/).

The novel taxa listed in Table 1 were all isolated from humans and animals, except for NCTC 14075 Paraburkholderia agricolaris, which was isolated originally from leaf litter in the United States. NCTC 14318 Eikenella exigua, NCTC 14180 Eikenella halliae, NCTC 14179 Eikenella longinqua, NCTC 14393 Enemella dayhoffiae, NCTC 14394 Enemella evansiae, NCTC 14359 Escherichia ruysiae, and NCTC 14396 Pseudoxanthomonas winnipegensis are all of human provenance. The remaining nine strains are of animal provenance, including isolates of horse, bat, sheep, alpaca, raccoon, Northern elephant seal, and California sea lion origin.

The descriptions of all strains were published in 2019 (*n* = 1), 2020 (*n* = 11), and 2021 (*n* = 5). The valid publication and description of NCTC 14393 Enemella dayhoffiae and NCTC 14394 Enemella evansiae (1) include the description of the novel genus *Enemella*. The publication of the valid description of NCTC 14359 Escherichia ruysiae constitutes the first novel species to be assigned to the genus since 2015 upon the valid publication of the description of Escherichia marmotae (2).

The four *Shigella* strains were all accessioned into the NCTC from the 1917 to 1954 Murray Collection of pre-antibiotic-era *Enterobacteriaceae* (3). NCTC 14120, NCTC 14146, and NCTC 14154 were all isolated before 1919, with NCTC 14123 dating from before 1938.

Four of the five AMR-associated Escherichia coli (NCTC 14376 to NCTC 14381) accessions are MC1000 strains genetically modified to harbor variants of functional isopropyl-β-d-thiogalactopyranoside (IPTG)-inducible *mcr* genes that confer resistance to the antibiotic colistin. In 2018, they were used to demonstrate the detection of *mcr*-mediated resistance to polymyxins using matrix-assisted laser desorption ionization–time of flight mass spectrometry (MALDI-TOF MS) (4). NCTC 14376 is an unmodified strain and does not carry an *mcr* gene. The Clostridioides difficile strain NCTC 14385 harbors a 7-kb plasmid, named pCD-METRO, which contributes to phenotypic metronidazole resistance to the strain (5), further elucidating the role of plasmids in metronidazole resistance in Clostridioides difficile.

NCTC 14169 and NCTC 14174 are Clostridioides difficile reference strains of PCR ribotypes 017 and 015, respectively. NCTC 14174 is the first PCR ribotype 015 isolate of Clostridioides difficile to be accessioned into the NCTC.

NCTC 14181 is an additional reference strain for *Eikenella exigua* (6), and NCTC 14178 is an additional reference strain for Auritidibacter ignavus (7). NCTC 14481 Legionella wadsworthii was isolated originally from a recent instance of human infection, reflecting the role of this species as a known but infrequent cause of legionellosis.

NCTC 14457 to NCTC 14465 are eight strains of Staphylococcus aureus that carry the methicillin resistance gene *mecC* isolated from human clinical sources (8). These strains were isolated originally in a six-hospital surveillance study in the United Kingdom and date from a little over a year after the *mecC* gene was first discovered in an isolate recovered from a milk bulk tank and consequently described (9).

NCTC 14405 to NCTC 14408 are four strains of Neisseria animaloris isolated from the wounds of stranded porpoises. They are described in a study elucidating the role of Neisseria animaloris infection following traumatic injuries by gray seals and the potential of Neisseria animaloris as a zoonotic pathogen from both animal species (10). They are the first known strains of Neisseria animaloris that were not isolated from cats, dogs, or humans (H. Malnick, personal communication).

The NCTC 14201 Ideonella sakaiensis type strain was isolated originally from a microbial consortium obtained from a poly(ethylene terephthalate) (PET; a humanmade plastic) bottle recycling site in Sakai, Japan, and can degrade and utilize PET as a carbon source (11). NCTC 14293 Varibaculum cambriense is also the type strain of its species, and its addition to the NCTC fortifies the variety of clinically relevant anaerobic species held by the collection (12). NCTC 14582 Escherichia coli is an additional strain of K-12 added to the NCTC, with the precedently held K-12 strain being NCTC 10538 Escherichia coli.

In conclusion, the UK National Collection of Type Cultures is a dynamic collection, and the accessioning of additional bacterial strains ensures the collection’s continuing relevancy to the work and activities of the scientific community. The collection enjoys collaboration and interaction with researchers worldwide, which is reflected in the newly accessioned bacterial strains described above.

The 47 strains described here represent modest growth in the holdings of the NCTC, and growth slowed in part due to the coronavirus disease 19 (COVID-19) pandemic. It is a requirement that in order to validly describe new bacterial taxa that the type culture is deposited in two recognized culture collections in two different countries (13), and the approximate third of the strains described above that serve as the type strains of novel taxa highlight the role of the NCTC in the description of novel bacterial taxa.

More information on depositing bacterial strains into the NCTC, a free service provided globally, is available on the NCTC/UKHSA Culture Collections website at https://www.culturecollections.org.uk.

### Data availability.

The full details for each strain (e.g., citations for the full taxon descriptions, strain provenance, and validated growth conditions) can be obtained from the NCTC online catalogue. Links to the catalogue entry for each strain can be found in Table 1, as can the links to the requisite genomic data wherever they are available.
